# The Buoyancy of *Cryptococcus neoformans* Is Affected by Capsule Size

**DOI:** 10.1128/mSphere.00534-18

**Published:** 2018-11-07

**Authors:** Raghav Vij, Radames J. B. Cordero, Arturo Casadevall

**Affiliations:** a Department of Molecular Microbiology and Immunology, Johns Hopkins Bloomberg School of Public Health, Baltimore, Maryland, USA; Duke University Medical Center

**Keywords:** *Cryptococcus neoformans*, buoyancy, capsular polysaccharide, yeast density

## Abstract

Cryptococcus neoformans is an environmental pathogenic fungus with a worldwide geographical distribution that is responsible for hundreds of thousands of human cryptococcosis cases each year. During infection, the yeast undergoes a morphological transformation involving capsular enlargement that increases microbial volume. To understand the factors that play a role in environmental dispersal of C. neoformans and C. gattii, we evaluated the cell density of *Cryptococcus* using Percoll isopycnic gradients. We found differences in the cell densities of strains belonging to C. neoformans and C. gattii species complexes. The buoyancy of C. neoformans strains varied depending on growth medium. In minimal medium, the cryptococcal capsule made a major contribution to the cell density such that cells with larger capsules had lower density than those with smaller capsules. Removing the capsule, by chemical or mechanical methods, increased the C. neoformans cell density and reduced buoyancy. Melanization of the C. neoformans cell wall, which also contributes to virulence, produced a small but consistent increase in cell density. Encapsulated C. neoformans sedimented much more slowly in seawater as its density approached the density of water. Our results suggest a new function for the capsule whereby it can function as a flotation device to facilitate transport and dispersion in aqueous fluids.

**IMPORTANCE** The buoyancy of a microbial cell is an important physical characteristic that may affect its transportability in fluids and interactions with tissues during infection. The polysaccharide capsule surrounding C. neoformans is required for infection and dissemination in the host. Our results indicate that the capsule has a significant effect on reducing cryptococcal cell density, altering its sedimentation in seawater. Modulation of microbial cell density via encapsulation may facilitate dispersal for other important encapsulated pathogens.

## Abstract (precis)

The buoyancy of a microbial cell is an important physical characteristic that may affect its transportability in fluids and interactions with tissues during infection. The polysaccharide capsule surrounding C. neoformans is required for infection and dissemination in the host. Our results indicate that the capsule has a significant effect on reducing cryptococcal cell density, altering its sedimentation in seawater. Modulation of microbial cell density via encapsulation may facilitate dispersal for other important encapsulated pathogens.

## INTRODUCTION

Cryptococcus neoformans and C. gattii species complexes are important fungal pathogens that can cause pulmonary and meningeal disease in humans (1). In the environment, C. neoformans is commonly found in soil associated with pigeon excreta, while C. gattii is most commonly found on trees (2, 3). C. gattii isolates have been collected from marine and fresh water environments (4, 5). Cryptococcal infection occurs via the respiratory tract, where yeast particulates can colonize the lungs (6, 7). In immunocompromised patients, C. neoformans can disseminate from the lungs to other parts of the body, including the central nervous system, by crossing the blood brain barrier. The dissemination of C. neoformans yeast cells from the lung to the brain is critical in the development of meningeal disease. The yeast cells can undergo drastic morphological changes that allow the pathogen to evade host immune response. For instance, yeast cells can modulate capsule and cell body dimensions in response to environmental conditions such that cell dimensions can range from 1 to 100 µm in diameter (8–11).

The polysaccharide (PS) capsule is composed mostly of water (12). It is formed by a porous matrix of branched heteropolysaccharides, mainly glucuronoxylomannan, that extends radially from the cell wall (13). Capsule synthesis is induced under certain stressful conditions and provides protection against host defense mechanisms by acting as a physical barrier, interfering with phagocytosis and sequestering reactive oxygen species (ROS) and drugs (14, 15). The capsule is essential for the virulence of C. neoformans and is of interest for both therapeutic and diagnostic strategies (16).

Melanin is another important virulence factor, such that strains that lack the ability to melanize are less pathogenic (16). Melanin is formed by the polymerization of aromatic and/or phenolic compounds, including l-3,4-dihydroxyphenylalanine (l-DOPA), methyl-DOPA, and epinephrine or norepinephrine (17). In the presence of catecholamine precursors found in the human brain, *Cryptococcus* melanizes its inner cell wall (18). Melanized C. neoformans cells are found in the environment (19) and during mammalian infection (20), suggesting an important role of the pigment in C. neoformans biology and pathogenesis. Melanization protects cells against a variety of host immune mechanisms and antifungal drugs, as well as against radiation, desiccation, ROS, and temperature stress (21, 22).

Both the polysaccharide capsule and melanin are complex structures that are difficult to study. Consequently, it is important to apply biophysical methodologies to gain new insights into the physicochemical properties and biological functions of these major virulence factors (23). One such property that has not been studied in cryptococcal biology is cellular density, presumably a highly regulated characteristic that may reflect the physiological state of the cell under different conditions (24).

In the first century B.C., Roman writer Vitruvius described a “eureka” moment that the Greek polymath Archimedes had when, allegedly, he observed the displacement of water as he sat in a bathtub, which led him to establish the law of buoyancy (25, 26). In a biological context, Archimedes’ law (law of buoyancy) can be applied to calculate the ratio of the absolute mass and volume of an organism which could determine whether it floats or sinks in a fluid of given density. During centrifugation in a continuous Percoll density gradient, cells equilibrate upon reaching the point at which the gradient’s density matches their own. This allows us to estimate cell density of C. neoformans and C. gattii against bead standards of fixed density.

Cell density is used for the separation of cell populations, but the factors regulating cell density in microbiology remain understudied, despite the important role that it may play in the migration and dissemination of microbial and mammalian cells in fluids. This could be because the cell density depends on many biological and physical factors, which are often difficult to disentangle. Earlier studies found that cell density was affected by the osmolality of the medium in which the cells are grown (27, 28), the encapsulation of bacteria by polysaccharide capsule (29), and the cell cycle stage (30). Strains of Porphyromonas gingivalis with lower cell density were less susceptible to phagocytosis; however, this could be the result of the correlation between cell density and cell surface hydrophobicity (31). Other studies have also reported differences in cell density among different strains of mycobacteria and *Burkholderia* spp. (32, 33). In the context of eukaryotes, Saccharomyces cerevisiae cell density varies at different stages of cell cycle (34), and quiescent S. cerevisiae cell populations can be separated out using density gradients in a stationary-phase culture of the yeast (35).

The cell density of C. neoformans and the factors that affect it have not been previously investigated. In this study, we used Percoll isopycnic gradients to study the effect of capsule induction, antibody (Ab) treatment, and melanization on the cell density of C. neoformans.

## RESULTS

### Comparison of C. neoformans and C. gattii cell densities.

**FIG 1:**
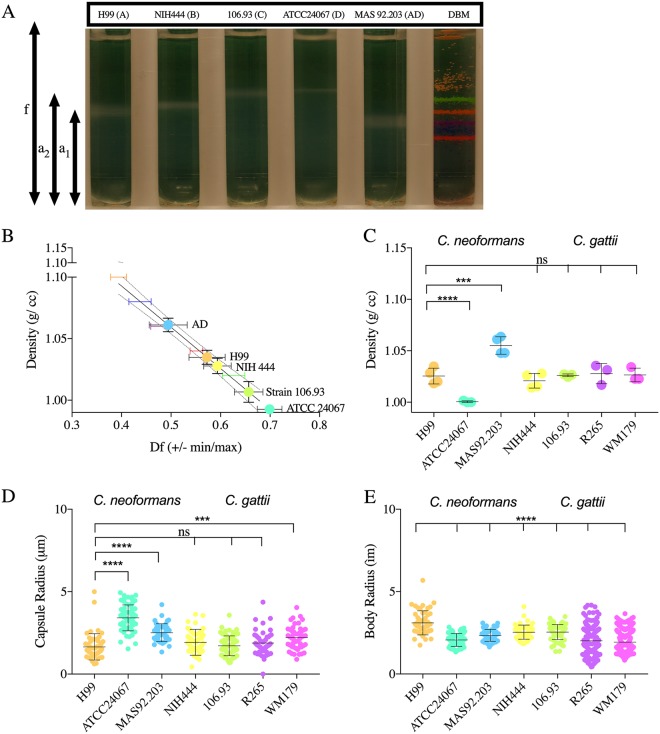
The cell density of C. neoformans and C. gattii serotypes. (A) Image representative of 3 to 4 independent repetitions of Percoll density gradients, comparing the cell densities of C. neoformans (serotypes A, D, and AD) and C. gattii (serotypes B and C) to that of density bead markers (DBM). (B) Representative data from 4 independent experiments, depicting the line interpolation of the density factor (minimum [min] and maximum [max]) calculated by pixel area per the following formulae: (*f* − *a*_1_/*f* and *f* − *a*_2_/*f*). The df (min, max) values of the density marker beads are used to estimate the cell density of the cells run in parallel gradients. (C) Histogram depicting the differences in cell densities of different serotypes of C. neoformans (serotypes A, AD, and D) and C. gattii (serotypes B and C and variants VGI and VGIIa). The experiment was performed 3 to 4 times, as indicated by the symbols on the bar graph. Error bars represent the SD about the mean. (D) Representative data of capsule (i) and cell body (ii) radii of different serotypes and strains (*n* = 3). Error bars represent the SD about the mean. One-way analysis of variance (ANOVA) was used for the comparisons of the cell densities and capsule and cell body radii of different strains and serotypes of C. neoformans and C. gattii to the respective measurements of strain H99 of C. neoformans. All comparisons were made to strain H99 of C. neoformans. The following symbols were used to annotate the statistical significance of the results: ns, *P* > 0.05; *, *P* ≤ 0.05; **, *P* ≤ 0.01; ***, *P* ≤ 0.001; ****, *P* ≤ 0.0001.

Cell densities differed consistently among different serotypes of C. neoformans and C. gattii species complex strains (Fig. 1C). The cell density of replicates showed significant variability in comparisons of C. neoformans serotype A (strain H99) to serotype D (strain ATCC 24067) and serotype AD (strain 92.903). C. gattii strains showed less variability than the C. neoformans isolates (Fig. 1C). To ascertain whether there was a relationship between cell density and cell dimension, we imaged the cells with an India ink counterstain and calculated both the capsule radii and cell body radii for the C. neoformans and C. gattii strains. We observed statistically significant differences in the cell body radii of all strains compared to C. neoformans serotype A (strain H99) (Fig. 1E). We also observed that the capsule radii of C. neoformans serotype D and AD and of C. gattii VG IIa were significantly different from those of serotype A (Fig. 1D).

### Effect of capsule induction on C. neoformans cell density.

**FIG 2:**
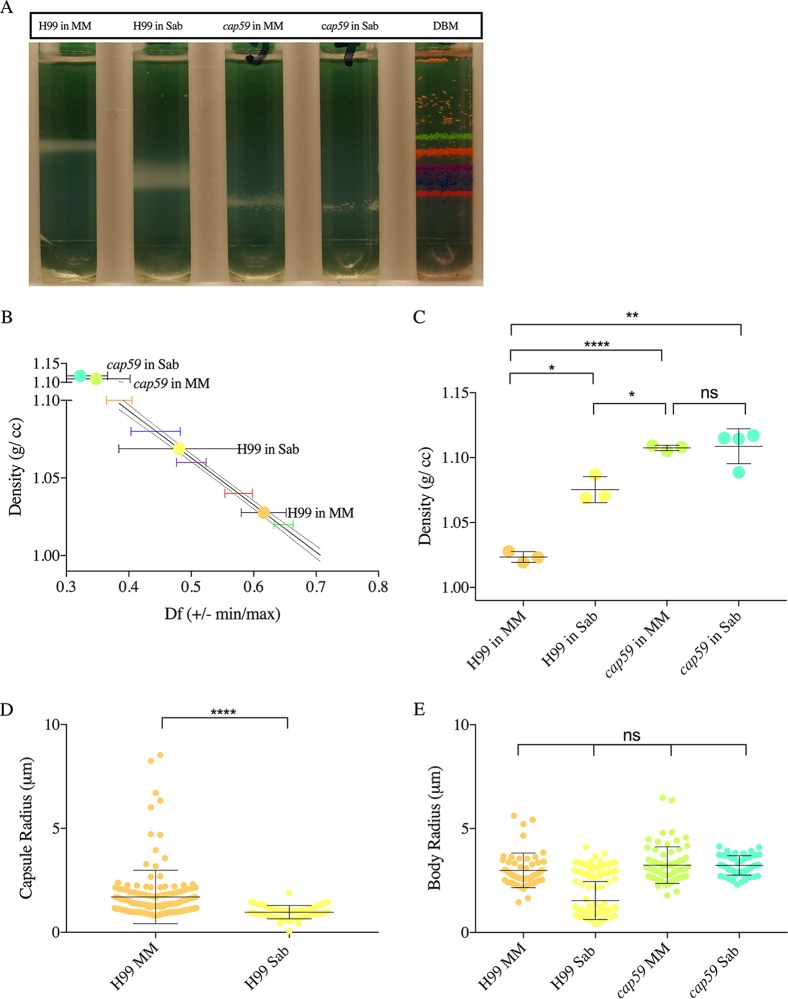
Induction of capsule synthesis decreases C. neoformans cell density. (A) Image representative of three independent repetitions of Percoll density gradients, showing the density of C. neoformans H99 juxtaposed with acapsular mutant *cap59*, both grown in Sab or MM. (B) Representative data from three independent experiments, depicting a line interpolation of the density factor with the cell densities of the bead standards to calculate the cell densities of the gradients run in parallel. (C) Histogram depicting a decrease in cell density of H99 cells grown in MM compared to Sab, due to capsule induction. *cap59* mutant cells are significantly denser than normal H99 cells grown in MM. Each data point on the histogram represents an independent replicate (*n* = 3); error bars represent SD about the mean. (D) Representative data depict (i) the capsule radii and (ii) the cell body radii of C. neoformans H99 and *cap59* grown under different medium conditions (MM or Sab) (*n* = 3). Strain *cap59* cells grown in MM and Sab do not have a capsule; therefore, the capsule radii were not quantified. Error bars represent SD about the mean. One-way ANOVA was used for the comparisons of cell density, capsule density, and cell body radii of C. neoformans *cap59* and H99 grown under different conditions. The following symbols were used to annotate the statistical significance of the results: ns, *P* > 0.05; *, *P* ≤ 0.05; **, *P* ≤ 0.01; ***, *P* ≤ 0.001; ****, *P* ≤ 0.0001.

*In vitro*, the capsule is induced under stress conditions such as exposure to nutrient starvation medium (36). Cells grown in minimal medium (MM) had significantly lower density (Fig. 2A to C) than cells grown under nutrient-rich conditions in Sabouraud dextrose broth (Sab) where the capsule was significantly smaller. Cells of the acapsular strain *cap59* had significantly higher density than encapsulated cells with the same genetic background. Furthermore, we observed no significant differences in the densities of acapsular mutants grown in minimal versus rich medium, confirming the contribution of the polysaccharide capsule in determining the cell density in response to different nutrient conditions.

**FIG 3:**
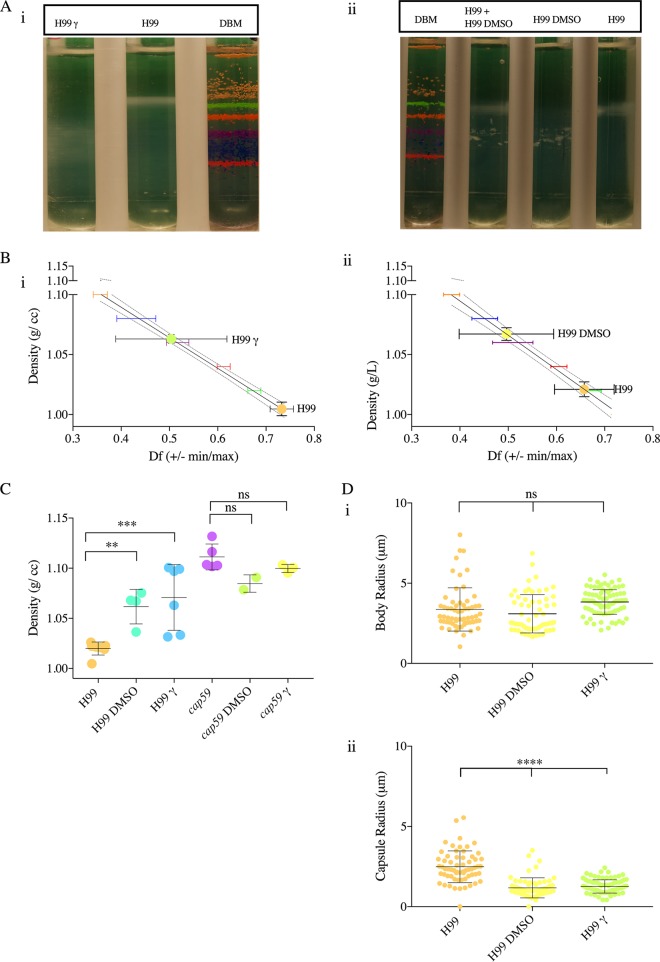
Removal of C. neoformans polysaccharide capsule increases cell density. (A) (i) Image representative of five independent repetitions of Percoll density gradients, comparing the cell densities of irradiated (γ) and nonirradiated C. neoformans (H99) grown in MM for 10 days with a standard of colored uniform density beads. (ii) Image representative of four independent Percoll density gradients of encapsulated C. neoformans H99 strains grown in MM for 10 days before and after DMSO extraction. (B) Representative data from independent experiments, depicting a line interpolation of the density factor (df) with the cell densities of the bead standards, to calculate the cell densities of C. neoformans before and after extraction of the capsule, run in parallel. (C) A histogram depicting cell density of C. neoformans before and after capsule extraction by γ rays and DMSO. The experiments were performed 3 to 4 times independently as indicated by the symbols on the bar graph; error bars represent SD about the mean. One-way ANOVA was used to determine differences between the densities of strains H99 and *cap59* grown in MM for 10 days with strains treated with DMSO and gamma radiation for capsule removal, respectively. (D) Representative data depict (i) the capsule radii and (ii) the cell body radii of C. neoformans before and after gamma irradiation capsule shedding and DMSO extraction (*n* = 3). Error bars represent SD about the mean. One-way ANOVA was used for the comparisons of cell densities and capsule densities and cell body radii of C. neoformans strain H99 to those of C. neoformans strain H99 after capsule removal by DMSO and gamma irradiation. The following symbols were used to annotate the statistical significance of the results: ns, *P* > 0.05; *, *P* ≤ 0.05; **, *P* ≤ 0.01; ***, *P* ≤ 0.001; ****, *P* ≤ 0.0001.

Previous studies have reported on the molecular composition of the C. neoformans capsular polysaccharide by studying the polysaccharide isolated from the cell surface by dimethyl sulfoxide (DMSO) extraction and gamma irradiation-induced capsule shedding (37). To confirm the effects of the capsule on the cell density, encapsulated H99 cells were treated with gamma radiation and DMSO to remove capsular material (Fig. 3). We observed a significant increase in cell density when the capsule was removed by both treatments, indicating that the polysaccharide capsule influences the cell density.

### Capsule size correlates with cell density.

**FIG 4:**
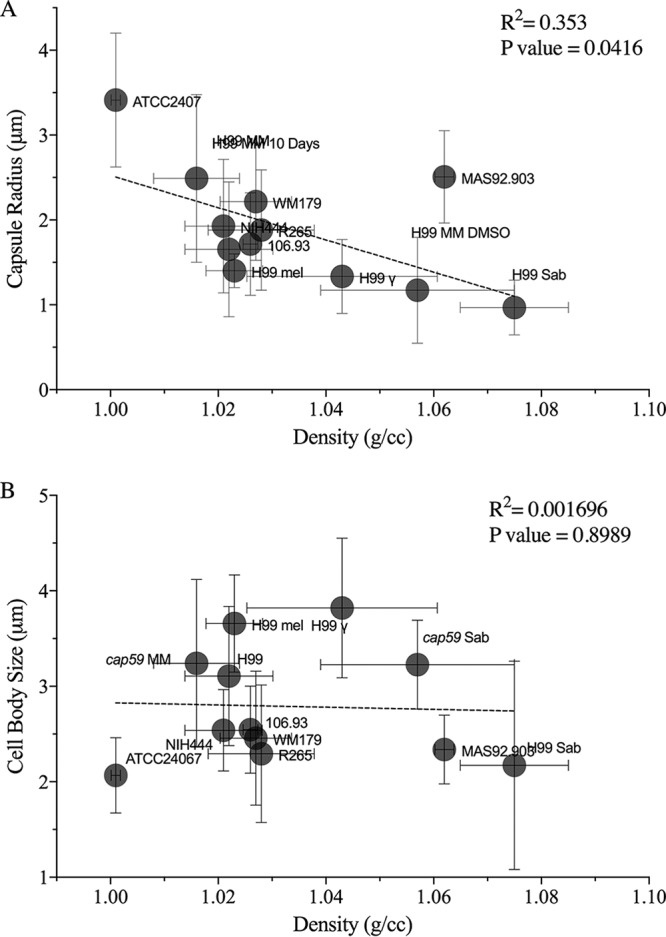
The densities of C. neoformans and C. gattii correlate with the capsule radii. (A) Density (in grams per cubic centimeter) significantly correlates with the capsule size (in micrometers). Data points are labeled such that, e.g., “H99 MM 10 days” represents C. neoformans strain H99 grown in MM for 10 days. (B) No linear relationship was found in comparisons of the cell body size data (radii) to the density data. The density values were collated from all the experiments performed under a specific condition (*n* = 3 to 5). The cell size data were taken from a single experiment corresponding to each condition whose results were found to be representative of the replicates.

Linear regression analysis revealed that the capsule radius correlates with cell density such that yeast cells with larger capsules had lower cell density (Fig. 4A). We observed some scatter in the linear relationship between capsule size and density, which presumably reflects other variables that may contribute to the density such as differences in lipid, glycogen content, and polysaccharide composition. There was no significant relationship between cell body size and cell density (Fig. 4B).

### Encapsulated Cryptococcus neoformans settles more slowly in seawater.

**FIG 5:**
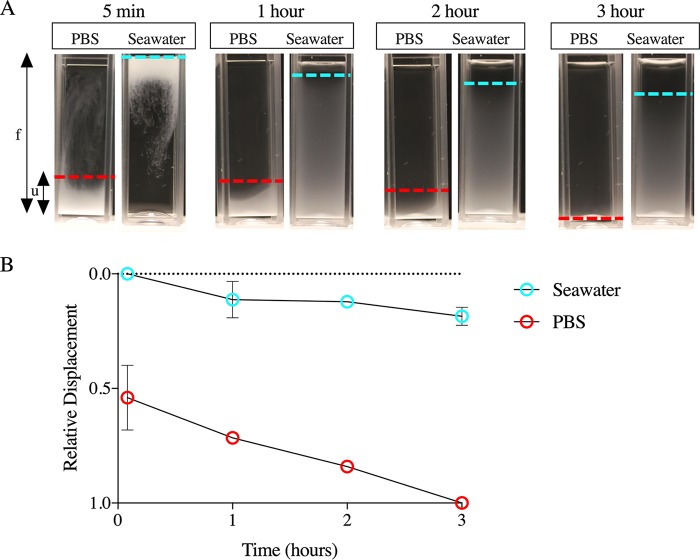
Encapsulated C. neoformans settles more slowly in seawater. (A) An image representative of cuvettes (3 ml) containing PBS (left) and seawater (right) imaged at different time points shows that H99 grown in MM settles faster in PBS. The relative displacement of cell sedimentation was calculated as (f − *u*)/*f*, where *f* is the area of the tube and *u* is the area from the bottom of the tube to the upper menisci of layers of settling cells. At 5 min, the relative displacement of cell sedimentation in seawater was 0, since all the cells were floating. In contrast, by 5 min, a large population of yeast cells suspended in PBS had already sedimented. At 3 h, the relative displacement value for sedimentation in PBS was 1, as all the cells had completely settled. (B) Plot of the normalized displacement of the upper menisci of cells settling in seawater and PBS. A line is drawn through the mean value of relative displacement at a given time intervals and the error bars represent standard deviation about the mean (*n* = 3 independent experiments). At certain time intervals, the error bar is smaller than the symbol and was not been plotted.

We tested whether the lower density of encapsulated C. neoformans allowed the fungi to float in water or seawater. The density of seawater is 1.0236 g/cc at room temperature (38, 39), and the density of C. neoformans in MM is 1.0220 ± 0.0082 g/cc. When C. neoformans grown in MM was added to a cuvette containing seawater, a large population of cells became suspended in the seawater, which became turbid (Fig. 5A). This effect was not seen with phosphate-buffered saline (PBS), where the cells sank to the bottom within 3 h (Fig. 5B).

### Melanization increases C. neoformans cell density.

**FIG 6:**
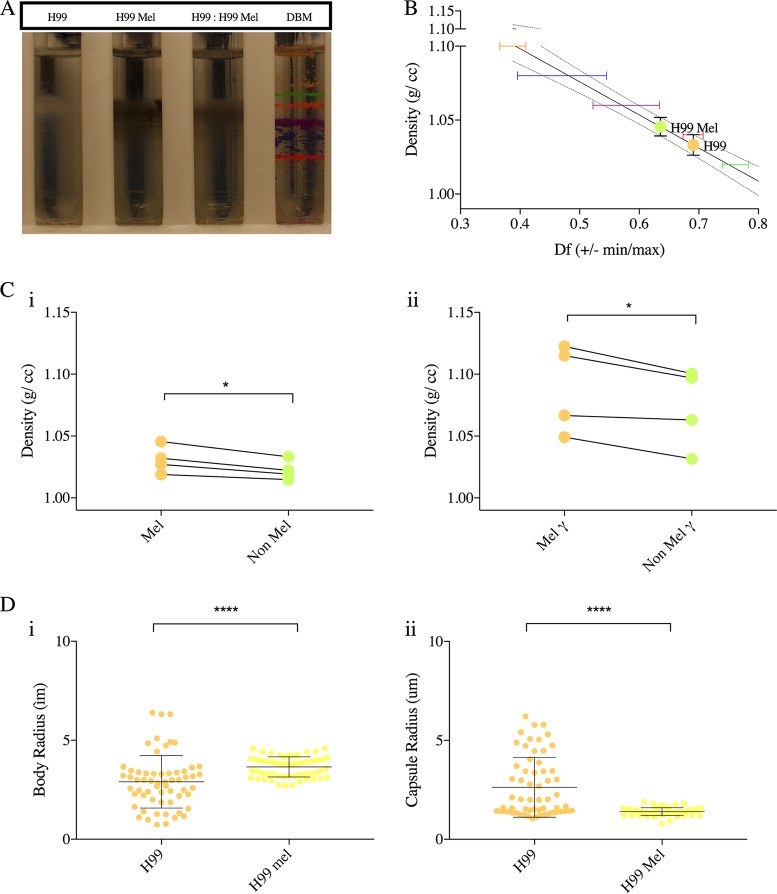
Effect of melanization on C. neoformans cell density. (A) Image representative of four independent repetitions of Percoll density gradients, comparing the densities of H99 in MM and of H99 in MM with l-DOPA (mel) and a 1:1 mixture of the cells. The white cells (H99) banded slightly above the melanized black cells (mel), as can be seen by the visualization of the gradient that contained the mixture. (B) Representative data from four independent experiments, depicting a line interpolation of the density factor with the cell densities of the bead standards, to calculate the cell densities of the gradients run in parallel. (C) (i) A histogram depicting density of melanized and nonmelanized C. neoformans to compare the densities of melanized and nonmelanized cultures started from the same Sab preculture. The experiment was performed using replicates (*n* = 4), as indicated by the symbols on the bar graph. A paired *t* test found the pairing results to be significant (**) and found consistent and significant differences (*) between nonmelanized and melanized cells. (ii) A histogram depicting the density of melanized and nonmelanized cells after removal of capsule by gamma radiation. The experiment was performed in pairs, such that the paired cultures were inoculated from the same Sab preculture and were treated with gamma radiation (1,500 Gy) together. A paired *t* test found the pairing results to be significant (*) and found consistent and significant differences (*) between nonmelanized and melanized cells treated with gamma radiation. (D) Representative data depict (i) the cell body radii and (ii) the capsule radii of melanized and nonmelanized C. neoformans cells (*n* = 2). Error bars represent SD about the mean. *t* test was used for the comparisons of capsule and cell body radii of melanized and nonmelanized cells of strain H99 of C. neoformans. The following symbols were used to annotate the statistical significance of the results: ns, *P* > 0.05; *, *P* ≤ 0.05; **, *P* ≤ 0.01; ***, *P* ≤ 0.001; ****, *P* ≤ 0.0001.

Comparison of melanized and nonmelanized H99 C. neoformans cells demonstrated that melanization was associated with a moderate increase in cell density (Fig. 6). Since the increase in the density was small and since melanized cells can easily be distinguished visually from nonmelanized cells, we mixed the melanized and nonmelanized cells in 1:1 ratio before loading the samples onto the density gradient. While nonmelanized cells displayed a range of density that overlapped that of melanized cells, the latter tended to have higher density than nonmelanized cells inoculated from the same Sab preculture. Isolated melanin “ghosts” (40) had much greater density than the cells, estimated to be >1.1 g/cc (data not shown). Note that melanized cells also had smaller capsules (Fig. 6D, panel ii), which might have contributed to the increase in cell density. Thus, we also compared the densities of melanized and nonmelanized cells after removal of the capsule by gamma radiation (Fig. 6C, panel ii). Upon capsule removal, we observed that the density of melanized cells was consistently and significantly higher than that of nonmelanized cells.

### Antibody binding and other conditions that have no significant effect on C. neoformans cell density.

Previous studies showed that capsular antibodies alter the viscoelastic properties and structure of the capsule (41). Antibody binding also causes a change in the hydration state of the PS capsule (12). Treatment of H99 C. neoformans with capsular antibodies (18B7 and E1) did not significantly alter the cell density (see Fig. S1 in the supplemental material). Furthermore, binding of mouse complement, incubations at different salt concentrations (to induce osmotic stress), and incubation in lipid-rich medium had no significant impact on C. neoformans cell density (Fig. S2).

10.1128/mSphere.00534-18.1 FIG S1 Binding of capsular antibodies does not alter C. neoformans cell density. (A) Image representative of independent repetitions of Percoll density gradients, comparing the densities of C. neoformans H99 with and without antibody incubation with (i) 18B7 and (ii) ES1. (B) Representative data from independent experiments, depicting a line interpolation of the density factor with the cell densities of the bead standards to calculate the cell densities of the gradients run in parallel. (C) A histogram depicting density of C. neoformans H99 incubated with capsular antibodies. Data represent capsular antibodies (i) 18B7 (*n* = 4) and (ii) ES1 (*n* = 2) at different concentrations (0.1, 1, 10, and 20 μg/ml). Download FIG S1, TIF file, 1.7 MB. Copyright © 2018 Vij et al. 2018 Vij et al. This content is distributed under the terms of the Creative Commons Attribution 4.0 International license.

10.1128/mSphere.00534-18.2 FIG S2 Effect of complement binding, osmotic shock, melanin-binding antibody, and growth in rich lipid media on cell density. Images of Percoll density gradients are presented to compare the densities of C. neoformans H99 upon (A) complement binding, (B) osmotic shock, (C) 6D2 antibody binding, and (D) growth in lipid-rich media. These conditions did not affect the cell density of Cryptococcus neoformans (H99) significantly. Experiments were done once. Download FIG S2, TIF file, 2.0 MB. Copyright © 2018 Vij et al. 2018 Vij et al. This content is distributed under the terms of the Creative Commons Attribution 4.0 International license.

## DISCUSSION

In this study, we characterized the cell densities of C. neoformans and C. gattii under different conditions. We report minor differences in cell densities between serotypes of the cryptococcal species complex. Our results also suggest that the capsule plays a major role in determining the cell density of the yeast such that encapsulated strains have densities close to that of water. Meanwhile, melanization increased the density slightly. Changes in buoyancy could influence the dispersal of the yeast in the environment and dissemination of the fungal pathogen during infection. Furthermore, the cell density may be used for the separation of different populations of yeast cells (35) and to characterize C. neoformans mutants with capsular defects (16) and for the isolation of the titan cells (42).

The cell density of a microbe is a fundamental biophysical property that influences its behavior in aqueous fluids. Depending on its density, a microbe could remain suspended in a fluid or settle to the bottom. Among other factors, this could influence the microbe’s access to nutrients, sunlight, and oxygen. Thus, it is not surprising that marine and freshwater unicellular organisms including phytoplankton, regulate their cell density via mechanisms that involve the synthesis and storage of gas vacuoles, polysaccharide mucilage sheaths, and glycogen (43). Interestingly, the polysaccharide mucilage sheath of these bacteria, which resembles the polysaccharide capsule of C. neoformans, has been characterized as an important factor that decreases the density of the cell to just about the density of water (44). Our data demonstrate that the cryptococcal capsule serves a similar function by increasing the volume of the yeast cell without significantly increasing its mass and thereby reducing its density.

A quantitative parameter used to determine how fast a population of microbial cells sinks in a fluid of given density is the settling velocity, which is calculated by the Stoke’s law and depends on the cell density and the size (diameter) for a spherical object such as a yeast cell (43, 45). In marine bacteria, low cell density (<1.064 g/cc) correlates with low settling velocity as calculated by Stoke’s law (46). The variable size (3 to 16 μm in diameter) of C. neoformans grown in MM and the low density (∼1.022 g/cc) that we observed under nutrient starvation conditions in an aqueous environment suggest that the settling velocity of C. neoformans would be similarly low. More importantly, the encapsulated C. neoformans cells would have a lower settling velocity than similarly sized cells that have no capsule, due to the decrease in density.

We hypothesize that the capsule can function as a flotation device that allows the yeast cell to float and move along with currents in aqueous environment to access nutrients and oxygen (47) and may facilitate the dispersal of the pathogen (48). For instance, a previous study found that a C. gattii clinical isolate survived in filtered ocean water, distilled water, and saline water (up to 10% of initial inoculum) at room temperature for up to 94 days (49). The resistance of *Cryptococcus* to different levels of osmotic stress is consistent with our observations showing that high salt concentrations do not alter cell density. The strains of C. neoformans and C. gattii have heterogeneous global distribution, and the mechanisms of the dispersal are unknown (50). Possibly, the various densities of the strains influence the differential dispersal of the fungal pathogen. Thus, in the context of environmental fungal pathogens C. neoformans and C. gattii, the cell density could play an important role in determining the dispersal of the yeast in the environment and could affect its ability to infect a wide range of hosts, including marine mammals such as dolphins (51–53). Estimating the settling velocity of microbial cells in aqueous fluids will add weight to the hypothesis that the cell density of C. neoformans and C. gattii influences environmental dispersal.

We also examined the effect of melanin, another major virulence factor, on the density of C. neoformans. Melanization had a moderate influence on cell density. Despite the much greater density of melanin ghosts, cellular melanization had a small effect on cell density, presumably due to the fact that melanin contributes only approximately 15.4% (mass/mass) of cellular mass (40).

In immunocompromised hosts, C. neoformans can disseminate from the lungs to the brain, where it causes life-threatening meningitis. This multistep process could require the fungal cell to travel into the draining lymph node and into fluidic blood and lymph systems to survive and grow outside the lungs. Murine models have shown that capsule and cell body sizes are different at different sites of infection (9). Our results show that capsule induction decreased cell density and increased flotation. Thus, capsular enlargement may be a variable that determines the dissemination of C. neoformans in body fluids.

In summary, the density of C. neoformans grown in MM is slightly greater than that of water. The presence of a capsule reduced the density such that it approached that of water. Hence, the capsule, by reducing density, also reduces the settling velocity of C. neoformans in aqueous solutions, which could favor environmental dispersal. The establishment of C. gattii in the Pacific Northwest is reported to have occurred relatively recently (50). Clinical isolates of C. gattii have been recovered from North America, although the means by which they reached the continent remain unknown (49). Our finding that yeast cells have low cell density and a propensity to float suggests that sea currents could have transported the pathogen between continents. The observation that the polysaccharide capsule makes a large contribution to reducing density suggests a new role for this structure in the environment as an aid to cell dispersal and transport in aqueous fluids.

## MATERIALS AND METHODS

### Yeast cultures.

Frozen stocks of C. neoformans and C. gattii strains were inoculated into Sab (pH adjusted to 7.4) at 30°C for 48 h. The C. neoformans and C. gattii strains used for this manuscript are described in Table 1. Approximately 10^6^ cells from the stationary-phase cultures in Sab were washed twice in MM (10 mM MgSO_4_, 29.3 mM KH_2_PO_4_, 13 mM glycine, 3 µM thiamine-HCl, and 15 mM dextrose, with pH adjusted to 5.5). The washed cells were inoculated into MM for capsule induction, MM with l-DOPA (100 mM) to induce melanization, and Sab for rich medium conditions. Cells were incubated at 30°C for 48 h with rotation at 180 rpm. Cells were washed twice with sterile PBS and were centrifuged for 5 min at 4,700 × *g*. Cells were counted using a hemocytometer, and dilutions were made to obtain 1 × 10^7^ cells in PBS. The cells were then loaded onto Percoll density gradients with or without treatments to test the effect of different conditions on the cell density.

**TABLE 1:** List of *C. neoformans* and *C. gattii* strains used

Species	Strain(s)	Reference or source
*C. neoformans*	H99	John Perfect (Durham, NC)
	*cap59*	54
	ATCC 24067	ATCC (Manassas, VA)

*C. neoformans* hybrids	MAS92-203	55

*C. gattii*	NIH444, ATCC 32609	55
	106.93	55
	VGI, WM179	ATCC (Manassas, VA)
	VGIIa, R265	ATCC (Manassas, VA)

### Density gradient centrifugation.

Percoll is a nontoxic and isotonic alternative to the commonly used sucrose gradient and is composed of polyvinylpyrrolidone-coated colloidal silica particles (56). Percoll has found applications for separation of mammalian blood cells, tumor cells, immune and endothelial cells, and microbial cells due to its ability to form reproducible self-generated continuous gradients (57). Stock isotonic Percoll (SIP) was obtained by addition of 1 part of 1.5 M NaCl to 9 parts of Percoll. The 70% (vol/vol) working solution was obtained by diluting SIP with 0.15 M NaCl to a final density of 1.0914 g/ml. A 3-ml volume of this solution was loaded into polycarbonate ultracentrifuge tubes (13 by 51 mm). Approximately 10^7^ cells were pelleted at 4,700 × *g* and overlaid in layers directly or after treatment. All gradients were run in parallel with a standard tube.

For the preparation of the standard tube, 10 µl of each uniform density bead standard (Cospheric DMB kit), including light orange (orange polyethylene microspheres sized 250 to 300 μm, with density 1.00 g/cc), fluorescent green (1.02 g/cc), florescent orange (1.04 g/cc), florescent violet (1.06 g/cc), dark blue (1.08) and florescent red (1.099 g/cc), was loaded and mixed with the Percoll.

By adjusting the time and speed of centrifugation, it was found that the most optimal separation of the density gradient beads (which was taken as an indication for the most optimal continuous density gradient formed) occurred at 40,000 rpm for 30 min (acceleration, 9; deceleration, 0), using a TLA 100.3 fixed-angle rotor with an Optima TLX tabletop ultracentrifuge.

### Cell density estimation.

First, the images of the density gradient were taken under conditions of uniform light and shadow using an Nikon D3000 digital single-lens reflex (DSLR) camera (auto settings). Next, pixel area measurements were taken from the bottom of the tube to the area at the beginning of each band (*a*_1_) and ranging to the end of each band (*a*_2_) and to the upper meniscus of the tube (*f*). The density factor, *D_f_* (min, max) was computed in Microsoft Excel with the formula below.

$$D_{f}\;(\text{min},\text{max})=\left\{\left(\frac{f-a_{1}}{f}\right),\left(\frac{f-a_{2}}{f}\right)\right\}$$

The averages, *D_f_* averages (*D_f_* avg), and standard deviations (SD) along the mean were computed in Microsoft Excel. A standard curve was derived using *D_f_* avg and SD (*x* axis) and cell density (in grams per cubic centimeter) (*y* axis) of the density marker beads. A 95% confidence interval was used to interpolate the mean density values of sample cells around a standard deviation, which were run in parallel with the uniform density bead standards.

Although the results from the different Percoll gradient runs followed the same trend, the exact density values differed considerably. This can be attributed to pipetting errors or errors in measurement of density factors.

### Gamma irradiation of cells for capsule removal.

Gamma irradiation was used to remove the capsule as described earlier (58). Approximately 10^9^ cells of melanized and nonmelanized cells were plated on a 24-well plate. The cells were irradiated with a total dose of 1,500 Gy, using Shepherd Mark 1 at the SKCCC Experimental Irradiator Core at the Johns Hopkins University Sidney Kimmel Comprehensive Cancer Center. Cells were washed twice in PBS, and approximately 10^7^ cells were pelleted at 4,700 × *g* and loaded onto the gradient.

### DMSO extraction of C. neoformans capsule.

Approximately 10^7^ cells were incubated in 15 ml of DMSO at 30°C for 30 min to allow capsule extraction. The cells were washed thrice in 1× PBS, pelleted, and loaded onto the Percoll density gradient.

### Antibody coating of C. neoformans capsule.

Purified 18B7 and E1 antibodies (kindly provided by Francoise Dromer’s laboratory) were obtained from stock solutions kept at 4°C. The antibodies were serially diluted in PBS to concentrations of 20 µg, 10 µg, 1 µg, and 0.1 µg/ml. A pellet of 10^7^ cells was suspended with 1 ml of each Ab solution in Eppendorf tubes, subjected to vortex mixing, and incubated at 28°C on a rotating mixer for 1 h.

### C. neoformans melanization.

Frozen stocks of C. neoformans H99 were inoculated into Sab and incubated at 30°C for 48 h until the cultures reached stationary phase. The cells were counted using a hemocytometer. A total of 10^6^ cells/ml were inoculated into MM (10 mM MgSO_4_, 29.3 mM KH_2_PO_4_, 13 mM glycine, 3 µM thiamine-HCl, and 15 mM dextrose, with pH adjusted to 5.5) with and without l-DOPA (100 mM). The cells were cultured for 10 days at 30°C with rotating at 180 rpm. The cells were washed twice in PBS, and ∼10^7^ melanized and nonmelanized cells and a 1:1 mixture of melanized and nonmelanized cells were loaded onto the gradient. Melanin ghosts were prepared as described previously (40).

### Mouse complement deposition in C. neoformans.

Frozen stocks of guinea pig complement (1 mg/ml) were thawed. A pellet of 10^7^ cells was added and adjusted to 50% (500 g/ml), 20%, 10%, and 1% dilutions with PBS. The cells were incubated with complement for 1 h at 28°C in a rotating mixer.

### Providing C. neoformans with osmotic stress.

C. neoformans (∼10^8^ cells) were incubated with 1 ml of 10× PBS, 1× PBS, 0.1× PBS, and ultrapure distilled water (MilliQ) for 2 h 30 min in a rotating mixer at 28°C.

### Settling of C. neoformans in seawater.

A total of ∼10^7^ cells/ml of C. neoformans grown in MM were gently pipetted onto cuvettes containing 3 ml of seawater (Worldwide Imports AWW84130 Live Nutri Seawater) and PBS. The settling of the cells was observed by imaging the cuvettes at different time intervals with a Nikon D3000 DSLR camera. The images were analyzed using ImageJ. The relative levels of displacement were measured using the formula (*f* − *u*)/*f*, where *f* is the area of the tube and *u* is the area from the bottom of the tube to the upper menisci of cells that are settling.

### Cell imaging and yeast size measurements.

The cells were visualized and imaged with India ink negative staining under an Olympus AX70 microscope at ×20 magnification and ×40 magnification. The capsule and cell body sizes were estimated using automated measurement Python software (59) or by the use of ImageJ when cells were observed to be aggregated.

### Statistical analysis.

All statistical analysis was performed using GraphPad Prism 7.0 software. The density of cells was estimated by making a standard curve from beads of different densities, using linear regression to estimate the unknown values of a given sample with a 95% confidence interval. Details of the statistical tests applied are provided in the figure legends.
