# Supplementary figures and images for: The Buoyancy of Cryptococcus neoformans Is Affected by Capsule Size
Source: mSphere. 2018 Nov 7;3(6):e00534-18. doi: 10.1128/mSphere.00534-18 (PMC6222054; doi:10.1128/mSphere.00534-18)

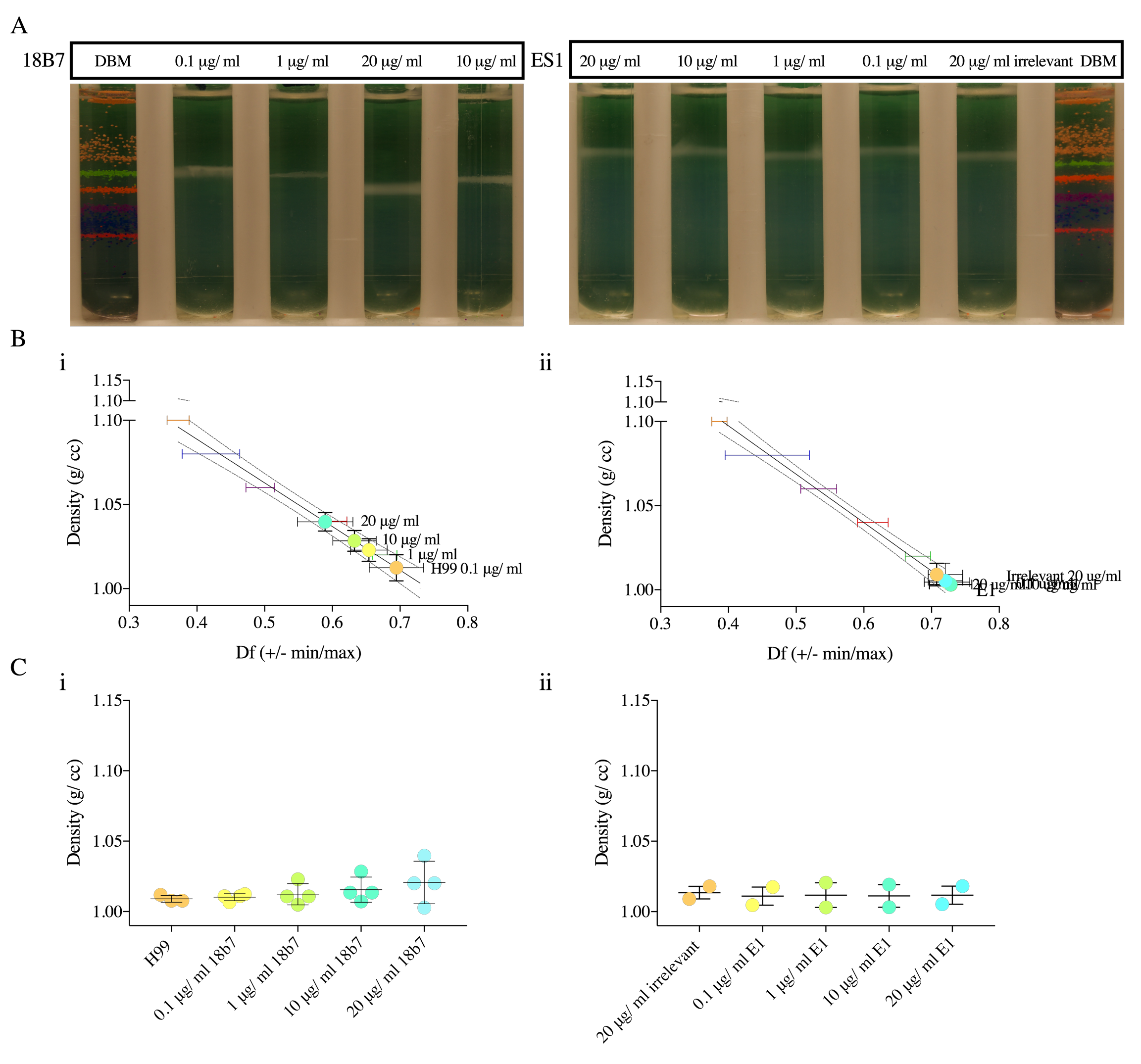

Supplement: FIG S1 [file sph006182697sf1.tif]

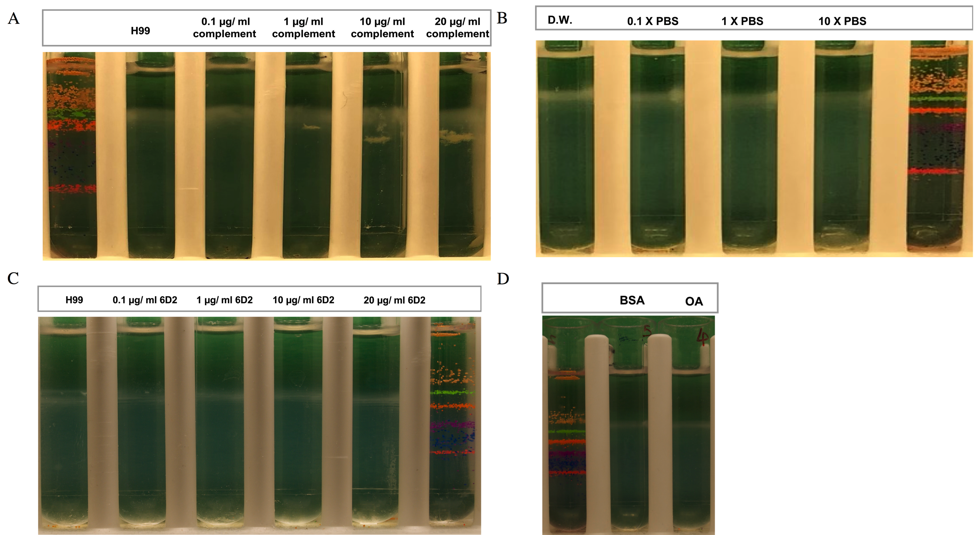

Supplement: FIG S2 [file sph006182697sf2.tif]
